# Opportunities and Challenges for Professionals in Psychiatry and Mental Health Care Using Digital Technologies During the COVID-19 Pandemic: Systematic Review

**DOI:** 10.2196/30359

**Published:** 2022-02-04

**Authors:** Hélène Kane, Jade Gourret Baumgart, Wissam El-Hage, Jocelyn Deloyer, Christine Maes, Marie-Clotilde Lebas, Donatella Marazziti, Johannes Thome, Laurence Fond-Harmant, Frédéric Denis

**Affiliations:** 1 Laboratoire Éducation, Éthique, Santé Université de Tours Tours France; 2 Centre d’Investigation Clinique Institut National de la Santé et de la Recherche Médicale Tours France; 3 Centre Hospitalier Régional Universitaire Tours Tours France; 4 Centre Neuropsychiatrique St-Martin Namur Belgium; 5 Département des Sciences de la Santé Publique et de la Motricité Haute Ecole de la Province de Namur Namur Belgium; 6 Department of Experimental and Clinical Medicine, Section of Psychiatry University of Pisa Pisa Italy; 7 Dipartimento di Medicina Clinica e Sperimentale, Section of Psychiatry University of Pisa, Unicamillus-Saint Camillus International University of Health Sciences Rome and Brain Research Foundation Lucca Italy; 8 Department of Psychiatry University of Rostock Rostock Germany; 9 Agence de Coopération Scientifique Europe-Afrique-Luxembourg Luxembourg Luxembourg; 10 Education et Pratiques en Santé Université Sorbonne Paris Nord Paris France

**Keywords:** COVID-19, e–mental health, professional practices, quality of care, telepsychiatry, videoconferencing

## Abstract

**Background:**

The COVID-19 pandemic has required psychiatric and mental health professionals to change their practices to reduce the risk of transmission of SARS-CoV-2, in particular by favoring remote monitoring and assessment via digital technologies.

**Objective:**

As part of a research project that was cofunded by the French National Research Agency (ARN) and the Centre-Val de Loire Region, the aim of this systematic literature review was to investigate how such uses of digital technologies have been developing.

**Methods:**

This systematic review was conducted following the PRISMA (Preferred Reporting Items for Systematic Reviews and Meta-Analyses) guidelines. The search was carried out in the MEDLINE (ie, PubMed) and Cairn databases, as well as in a platform specializing in mental health, Ascodocpsy. The search yielded 558 results for the year 2020. After applying inclusion and exclusion criteria, first on titles and abstracts and then on full texts, 61 articles were included.

**Results:**

The analysis of the literature revealed a heterogeneous integration of digital technologies, not only depending on countries, contexts, and local regulations, but also depending on the modalities of care. Notwithstanding these variations, the use of videoconferencing has developed significantly, affecting working conditions and therapeutic relationships. For many psychiatric and mental health professionals, the pandemic has been an opportunity to build up their experience of remote care and, thus, better identify the possibilities and limits of these digital technologies.

**Conclusions:**

New uses of such technologies essentially consist of a transition from the classic consultation model toward teleconsultation and make less use of the specific potential of artificial intelligence. As professionals were not prepared for these uses, they were confronted with practical difficulties and ethical questions, such as the place of digital technology in care, confidentiality and protection of personal data, and equity in access to care. The COVID-19 health crisis questions how the organization of health care integrates the possibilities offered by digital technology, in particular to promote the autonomy and empowerment of mental health service users.

## Introduction

The spread of digital technology in health systems is a major and irreversible phenomenon, a source of changes that are only just beginning. Initiated several decades ago in the field of psychiatry and mental health care, the development of digital technologies has been increasing for several years [1-3]. Teleconsultation has notably begun to be used in specific contexts, such as when access to health care is at stake for expatriates or people living in isolated areas, while remaining marginal [4,5]. Technical difficulties, concerns about confidentiality, and regulatory barriers are among the obstacles to the development of telepsychiatry [6]. The expansion of new technologies offers more and more possibilities, including sensors that are able to collect clinical data related to physical activity, stress, or sleep. Intelligent applications are able to detect changes in individual behavior and then analyze this data to assist in screening and monitoring mental illnesses. Not only do such technologies open up new possibilities, but they might also bring about decisive changes to enhance the overall efficiency of mental health services [1,3].

The COVID-19 pandemic has highlighted the potential of these technologies, which have led to digital uses on an unprecedented scale in psychiatry. In particular, the pandemic revealed the contributions of these technologies to ensuring continuity of care while annihilating the risk of viral transmission in the context of an outbreak. As they allow remote monitoring of some patients, these technologies have been used in a wide range of strategies to reduce the risk of transmission of SARS-CoV-2. They have also made it possible to carry out interventions responding to needs that are specifically related to the epidemic, whether it be support for frontline health professionals or care for patients with COVID-19. The use of teleconsultation, previously in mental health care and psychiatry in its early stages, has massively increased in response to the health crisis and among measures that have been implemented to contain it [7,8]. These experiences of telepsychiatry, which started in emergency situations and were facilitated by exceptional arrangements and, often, regulatory relaxation [9], raise many questions about the evolution of health care and involve ethical and regulatory issues [10].

Mental health and psychiatric care specifically provide a central place to the therapeutic relationship. In this context, our attention is focused on the impact of digital technologies as a “relational artifact“ (ie, the way they reconfigure care relationships).

The objectives of this study were (1) to describe the uses of digital technologies at the time of COVID-19 and their impact on professional practices in psychiatry and mental health and (2) to understand the place of digital technologies in the organizational adaptations linked to the COVID-19 epidemic, but also to identify how this specific context questions the modalities of care.

## Methods

This systematic review was conducted according to the PRISMA (Preferred Reporting Items for Systematic Reviews and Meta-Analyses) guidelines [11].

### Search Strategy

A systematic literature search was carried out in two databases, MEDLINE (ie, PubMed) and Cairn, and a specialized mental health platform, Ascodocpsy; all included articles met the inclusion criteria. Search terms were defined by articulating keywords, which were previously defined from dictionaries of synonyms and thesauri *alone* or *in combination with* the Boolean operators “AND” and ”OR” (Table 1). The search, which was carried out on titles and abstracts, only concerned the year 2020, with extraction of results taking place as of December 31, 2020. Our process did not generate any results in previous years, which is consistent with the date that the COVID-19 epidemic began. Only peer-reviewed articles accepted for publication and available in English or French were included. Editorials were excluded.

After a preliminary exploratory search, all authors agreed on the inclusion and exclusion criteria. To be included in the literature review, articles had to meet the following criteria: deal with the use of digital technologies as a response to the pandemic context and be related to the field of mental health care or psychiatry. On the other hand, the following were excluded: articles documenting the impacts of COVID-19 on mental health and psychiatry in general, adaptations of the health care offering carried out independently of the digital possibilities, and uses of digital tools in mental health and psychiatry independent of the COVID-19 outbreak.

In order to benefit from international experiences in an unprecedented context where many countries were simultaneously confronted with the same challenges, we chose not to exclude references based on geographical criteria.

**Table 1:** Search terms used to find articles for this review.

Database	Thesaurus	Search terms
PubMed (MEDLINE)	Yes	(“coronavirus” OR “covid-19” OR “sars-cov-2”) AND (“mental health worker” OR “psychiatry” OR “mental health professional” OR “psychiatrist” OR “psychologist” OR “psychiatric nurse” OR “e-professional in psychiatry” OR “e-mental health”)
Cairn	No	(“covid-19” OU “sars-cov-2” OU “coronavirus”) ET (“psychiatrie” OU “santé mentale” OU “psychologue” OU “infirmier en psychiatrie” OU “pair-aidant” OU “médiateur de santé pair” OU “e-professionnel de la psychiatrie”)
Base SantéPsy (Ascodocpsy)	Yes	Base set contains “covid-19” ET (“psychiatrie” OU “santé mentale” OU “psychologie” OU “hôpital psychiatrique”)

### Study Detection

**Figure 1:**
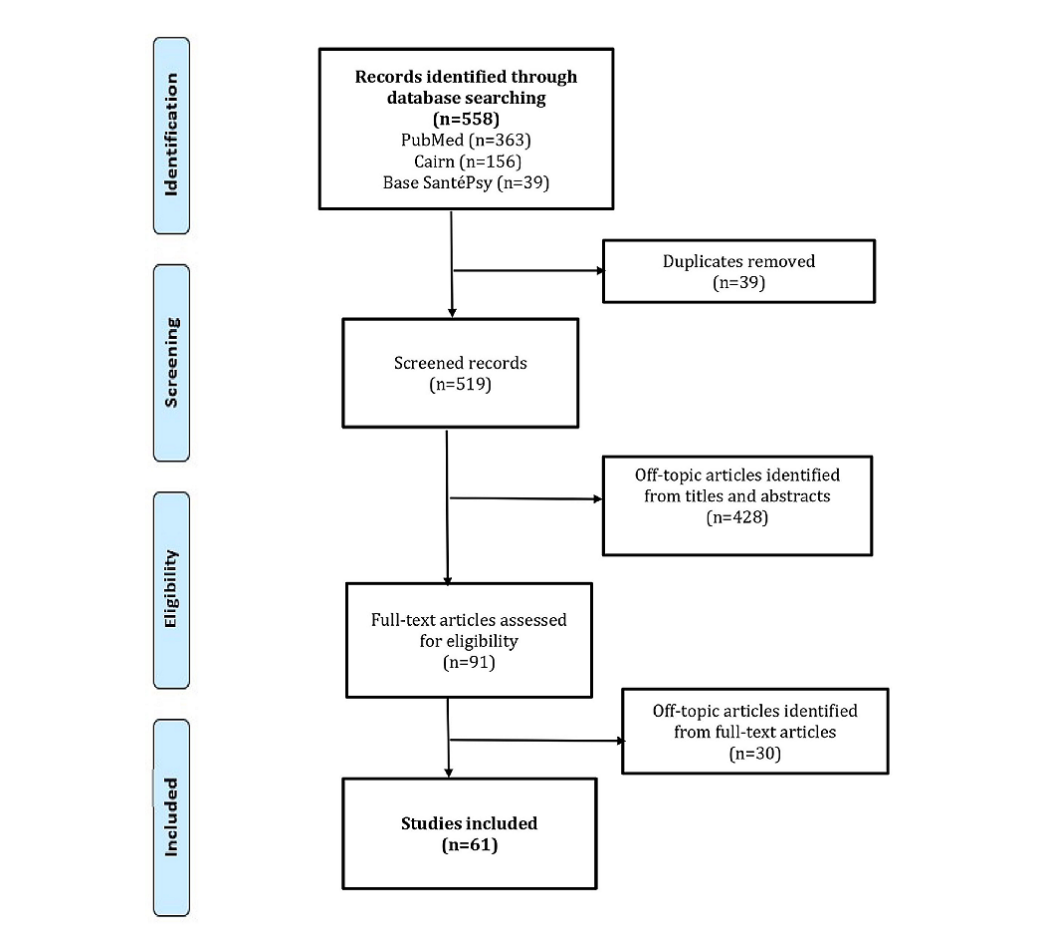
Flowchart of article selection for this review.

The search yielded a total of 558 documents, 39 of which were duplicates that were excluded. The first two authors (HK and JGB) preselected references by applying the inclusion and exclusion criteria on abstracts and agreed to select 91 articles. A careful reading of the documents resulted in the exclusion, after consultation, of 30 more articles. Therefore, a total of 61 references were selected (Figure 1).

## Results

### Contributions From the Use of Digital Technologies in Mental Health Care and Psychiatry

This literature review shows that, in the context of the current crisis and as professional practices need to adapt, publications have been produced at a rapid rate. The use of digital technologies appeared to be a crucial issue, which was addressed in 61 articles in the year 2020 alone. Table 2 [5,7,12-70] describes the characteristics of these articles, the digital technologies they addressed, their main uses, and the fields of application mentioned.

The methodological quality of these contributions turned out to be quite poor, due to a lack of time and hindsight to carry out more rigorous work. About half of them (30/61, 49%) were feedback articles. They show the willingness of those involved in psychiatry and mental health care to share their experiences and innovations in the midst of the COVID-19 crisis. Such publications reflect an acceleration in the exchange of professional practices on an international scale. They used diverse methodologies, ranging from personal narratives to more collaborative and structured forms of feedback and analysis of experience. The presence of 8 (13%) reflection-based articles also shows the willingness of professionals to share their concerns. Another set of 14 (23%) articles were literature reviews, either narrative or systematic. In the end, of the 61 articles, only 9 (15%) were original research studies.

Selected articles included many countries, spread over five continents, which were simultaneously confronted with similar issues related to the use of digital technologies in response to the pandemic. The largest number of articles concerned Western and Northern Europe (n=24, 39%) and North America (n=23, 38%).

**Table 2:** Characteristics and themes of the selected articles.

Characteristics		Studies (N=61), n (%)	References
**Type of article**			
	Experience feedback	30 (49)	[12-41]
	Literature review	14 (23)	[5,7,42-53]
	Study	9 (15)	[54-62]
	Reflection	8 (13)	[63-70]
**Location of study**			
	Europe	24 (39)	[5,13,14,18,19,26,27,29,31,33,36,38,40,42,44,50,54,57,58,60-62,64,65]
	North America	23 (38)	[7,12,15,17,20-24,30,32,34,35,37,39,41,45,46,48,49,52,63,66]
	Asia	8 (13)	[16,47,51,53,55,67,68,70]
	Australia	4 (7)	[25,43,59,69]
	Africa	2 (3)	[28,56]
**Digital tool**			
	Videoconferencing	45 (74)	[22,24,26-35,37,39-41,47,50,52,58,59,61,66,68-70]
	Telephone	27 (44)	[24-27,32,33,35-37,39,51,59,60,62,68,69]
	App	10 (16)	[29,47-49,51,53-55,64,67]
	Connected device	5 (8)	[47-49,65,67]
	Website	4 (7)	[30,53,56,68]
	Artificial intelligence	4 (7)	[48,49,64,67]
**Use of digital tools**			
	Patients’ follow-up and care	44 (72)	[5,7,12-21,23,25-29,31-38,41-43,46,48-50,52,57,58,60,61,63,65-68,70]
	Public support for COVID-19	9 (15)	[35,44,49,51,53,54,56,62,64]
	Group therapy	8 (13)	[17,19,21,30,34,39,50,67]
	Assessment and diagnosis	7 (11)	[22,25,45,49,60,62,65]
	Support for health professionals	5 (8)	[30,39,44,55,62]
	Care of patients with COVID-19	5 (8)	[14,16,17,40,53]
	Staff meeting	5 (8)	[7,16,27,34,41]
**Domain**			
	Psychiatry in general	29 (48)	[5,7,14,15,17,20,21,24,25,28,34,40-44,46,50,52,55,57-60,63,66-69]
	Psychology	14 (23)	[16,24,30,33,35,36,39,40,47,49,53,54,62,64]
	Mental health promotion	8 (13)	[44,47,49,51,53,56,62,64]
	Child psychiatry	5 (8)	[13,18,29,32,37]
	Geriatric psychiatry	5 (8)	[31,38,56,61,65]
	Community health	4 (7)	[12,23,27,68]
	Forensic psychiatry	3 (5)	[22,26,45]
	Addiction	3 (5)	[19,48,70]

### Adopting New Digital Technologies

Most of the articles selected for this literature review (45/61, 74%) mentioned the use of videoconferencing. This technology has been used, in particular, in interventions with mental health and psychiatric professionals who provide care for patients with COVID-19 [30,39,55] or, more broadly, for the population affected by the COVID-19 pandemic [40,51,62]. Videoconferencing consultations and group discussions took place alongside on-site interventions and telephone helplines.

However, teleconsultation has been massively developed to ensure continuity of care. Videoconferencing has made it possible to maintain not only remote monitoring of patients, but also therapy groups [17,48] and meetings between professionals. This particular use of this technology at an unprecedented scale has been developed at the intersection of two phenomena: the constraint of physical distancing measures aimed at containing the epidemic and the achievement of a high level of digital performance that allows for seamless use of videoconferencing. This rapid expansion of teleconsultation is perhaps the most important impact of the pandemic on the organization of care in psychiatry [7]. This sudden evolution has often gone along with the use of videoconferencing platforms, such as Skype, Zoom, or Microsoft Teams, despite their use raising security and confidentiality issues [17,20,21,32,41,43]. Specialized digital health platforms, such as MyChart by Epic, have been less frequently employed. In some cases, the use of videoconferencing has been combined with that of more familiar telecommunication tools, such as the telephone [17,36,38,57,59], emails, or SMS [34]. In our literature review, the telephone was the second most frequently mentioned digital technology, with 27 out of 61 (44%) references cited.

In most psychiatric services, however, this switch to remote communication is not yet complete, forcing practitioners to determine which activities require face-to-face meetings and which ones can be done via videoconferencing [14,16,23,25]. In particular, in-person examination has been maintained for patients who are deemed vulnerable and at risk [25].

As the number of remote consultations increased, prescription procedures have also been impacted. In order to limit the number of in-person appointments, practitioners have either used tele-prescription or opted for prescriptions covering a longer period. For treatments that require follow-up of specific clinical parameters, such as Clozapine, which involves monitoring blood counts, protocols have been made more flexible, sometimes allowing for a remote assessment of the clinical condition of patients [23,29,71].

In addition, 10 articles (16%) discussed connected apps and devices. In particular, their authors highlighted the relevance of connected apps and devices to assist remote monitoring during the pandemic [48,54,64]. Although the analyzed literature mentioned the potential of these new technologies, none of the identified publications documented the possible increase in their use in the context of a health crisis. Only 2 (3%) references were about apps that enabled the connection between patients and health professionals [51,55].

### Heterogeneous Integration of Digital Technologies

Although the COVID-19 pandemic has stimulated the development of telepsychiatry on all continents, our review of the literature allowed us to glimpse variations between countries. In the United States, due to the removal of regulatory barriers, the shift to telepsychiatry has been massive and even total in certain units, as illustrated by numerous publications [15,24,32,34,37,41]. In many European countries, telepsychiatry has gone from a niche practice to an essential modality for providing mental health services; this has been so in Germany [27], Spain [62], France [13,18,42], Ireland [19,29,50], the Netherlands [57], and Switzerland [65]. Telemedicine was adopted, even in countries such as North Macedonia, where public policies had previously been rather opposed to it [58]. A similar situation was reported in Australia [25,43,69]. In some cases, barriers persist, as in Canada, where the lack of health insurance coverage for teleconsultation with mental health professionals prevented its expansion during the pandemic [35]. Other countries, such as India, have been quick to innovate in favor of integrating digital technologies [47,68], and they rely on the development of telepsychiatry in order to increase health care delivery, despite limited resources. Developing telemedicine has been more difficult in some countries of the Global South due to a lower spread of information and communication technology [28,51]. Although advancing heterogeneously, the COVID-19 pandemic has been stimulating the integration of digital technologies into health care, confronting many countries simultaneously with comparable problems.

Within countries, these trends raise the issue of unequal access to digital technologies. Consequently, the development of telepsychiatry may disadvantage people living in poverty [23,63] and older adults who do not have access to these technologies [31,38,56].

Moreover, many authors have reassessed the appropriateness of telepsychiatry depending on the patients and their disorders, which had already been documented in the literature [3]. Telepsychiatry seems inappropriate for use with children whose attention is difficult to capture from a distance [29,69], as well as with older patients, who are less familiar with digital technologies and frequently suffer from hearing and visual impairments [38,60,61]. On the other hand, telepsychiatry is likely to facilitate access to health care for youth who are accustomed to new technologies [32].

Specific problems with the use of these technologies have arisen in certain fields, such as forensic psychiatry [45,72], the treatment of drug addiction [70], or electrostimulation techniques [7]. Although facilitating some procedures [22], remote forensic assessments are at risk of being disqualified for “procedural defects” [45]. On the other hand, the possibility of appearing in court by videoconference has prevented some forms of stress for people with mental disorders [26]. With regard to the treatment of drug addiction, specific difficulties may be related to legislative measures taken to prevent the diversion and misuse of certain drugs, by prohibiting tele-prescription, among other restrictions [70].

These numerous contributions found in the scientific literature, which were based on new experiments in the context of the COVID-19 pandemic, have added to the established knowledge about the relevance of telepsychiatry in different situations.

### Experiencing New Conditions of Professional Practice

The use of telepsychiatry, which makes it possible to reduce the risks of infection, has generated new conditions of practice for many professionals, defining both new possibilities and constraints. For independent practitioners, teleconsultation is no longer necessarily a freely chosen practice [4], but a means of maintaining their practice despite the restrictions imposed by public health measures. In hospital services, the decision to switch some services to a remote mode has been taken by those in charge in a more or less constrained way, or more or less consensually. Many professionals have had to adapt their practices, even though they were initially hostile to the use of digital technologies [5,42].

It should be noted that this new digital work experience has sometimes been associated with teleworking from home [27,33], in a general context of lack of preparation. Most of the professionals concerned were not trained in telepsychiatry follow-up, and some were not very comfortable with new technologies [33]. At the same time, they had to learn how to use digital technologies to support their patients, manage their own stress, and sometimes set up home-based work processes [15,52]. The accumulation of all these tensions can lead to emotional exhaustion [52].

Professionals teleworking from home have been confronted with unprecedented situations of temporal and spatial juxtaposition of both their professional and personal lives. This juxtaposition requires “psychological work to differentiate between private and professional lives that is more costly than usual” [33]. However, other authors mention that telework can also facilitate work-life balance in psychiatry [41,60], and practitioners reported that caring for a patient while teleworking nevertheless had a positive impact on their well-being in the midst of the crisis [41].

Moreover, the use of digital technologies, especially as their use is improvised and unframed, is likely to lead to an increased workload. Professionals may be exposed to an accumulation of requests through multiple technologies: videoconferencing, telephone, email, and SMS [15]. For them, the extensive use of videoconferencing can be a source of fatigue [24,52], described as “Zoom fatigue” [34], and a source of stress [34,52]. This fatigue is related to efforts to communicate and establish a relationship via videoconferencing [15], to the difficulty of sticking to a schedule with time slots that are explicitly dedicated to each patient, and to all the operations required to disconnect and reconnect to each device [34]. And yet, after overcoming technical and organizational obstacles, professionals can take advantage of those digital technologies, which can also bring more flexibility and help them save time [57], as documented in research prior to the pandemic [73].

Having experiences of care relationships reshaped by digital technologies in the context of this pandemic, psychiatric and mental health professionals have been using videoconferencing and the telephone to follow up on many patients. For health care providers, the COVID-19 crisis has been an opportunity to build up their experience of remote health care monitoring and, thus, better understand the possibilities and limitations of such digital technologies. This unprecedented context forced them to reinvent “relational mental health” [42]. Among other consequences, telepsychiatry favors more fragmented care modalities, with shorter and more frequent encounters [15,57].

Furthermore, although the effectiveness of telepsychiatry had already been documented [3,50,74], many professionals were skeptical about the possibility of establishing, maintaining, and strengthening a therapeutic relationship [66]. They feared that they would lose not only human contact, but also control over their image [3]. Some feared that the screen would become a barrier to the therapeutic process [29]. After experiencing telepsychiatry in the context of the health crisis, assessments remain contrasted [20]. Some authors mentioned that teleconsultation tends to hinder verbal and nonverbal communication [20,30,34,48,53]. In addition to the difficulty in grasping nonverbal body language [20,34,53], it is no longer possible to smell odors [46,53], see how patients are dressed, and perceive certain attitudes [46]. Communication must do without physical contact, such as greeting each other with a handshake [29], and empathy can no longer be manifested by comforting gestures [61]. Videoconferencing introduces a new mode of presence to the other, sometimes inducing a feeling of dissonance due to audiovisual presence and bodily absence [20]. The feeling of intimacy and confidence is not the same as in a closed office, and consultation no longer benefits from a separate space and time but is embedded in everyday life [30].

The professionals were also led to discover the advantages of digital technologies. The use of videoconferencing can be an opportunity to better contextualize some information, since part of the patient’s environment is made visible [16,53]. Teleconsultation can also make it possible to remove certain inhibitions and to access the unconscious more easily [9]. A few authors identified advantages of telephone consultations over videoconferences, especially for short talks [55]. In some cases, conducting telephone consultations allowed patients greater freedom of expression and allowed professionals to listen more carefully [14].

Although telepsychiatry allows for a large number of follow-up consultations, several authors mentioned that the greatest difficulty was in establishing a therapeutic relationship without a prior face-to-face encounter [46]. As a result, in the context of COVID-19, psychiatric and mental health professionals tended to postpone work on trauma, focusing instead on maintaining the patient’s well-being and encouraging activities to achieve this goal [34]. With regard to diagnosis, almost all professionals interviewed in a study that was conducted in Ireland reported that they were less comfortable making a diagnosis based on a telephone consultation [56]. Similarly, conducting neuropsychological assessments from a distance presents specific difficulties [14,61].

Although already documented, the possibilities and limits of telepsychiatry were highlighted by the COVID-19 epidemic, illustrating how experience can help to gradually shape new therapeutic practices integrating digital technologies.

## Discussion

### Principal Findings

The profusion of articles identified in the framework of this literature review shows how much the COVID-19 crisis has raised issues about care practices in psychiatry and how they integrate the available digital technologies. Such integration proves to be heterogeneous, depending on local contexts and regulations, but also regarding the fields and modalities of care. The use of videoconferencing has had an impact not only on the working conditions of mental health and psychiatric professionals, but also on the care relationships they maintain with their patients. This sudden shift to remote care has prompted professionals to publish papers about their experiences with telepsychiatry, sometimes in a naive way, without building on pre-existing research.

### Lack of Preparation Confronting Professionals With Ethical Questions

The experience of videoconferencing, in a context where mental health and psychiatric professionals had not been prepared for it, calls into question the quality of care [60]. Many professionals have used teleconsultation without training or knowledge of existing protocols and recommendations [75]. This lack of preparation pushed them to improvise and confronted them with ethical dilemmas. This unprecedented situation raises questions about privacy and the protection of personal data [47] as well as the risk of increasing inequalities in access to health care [48,50,63]. Indeed, the rapid expansion of telemedicine hinders access to health care for patients who do not possess, nor are proficient in, the necessary technology [9].

To guarantee the best conditions for confidentiality, special attention should be paid to the choice of digital technologies to be used. In the context of the current crisis, this choice has been little considered and is essentially based on pragmatic considerations. The use of new technologies requires special precautions, such as using headphones, consulting in a closed room, and disconnecting when absent [20]. Therefore, their use requires awareness and support from professionals in order to ensure the protection of personal data [47]. In addition, remote intervention involves knowing where the patient is and what local resources are available to respond to emergency situations [75].

The partial or total shift to remote consultations also raises the issue of equity in the provision of health care. Many professionals have been forced to identify vulnerable patients who require face-to-face encounters and those who can be monitored remotely [14,15,23,25]. This process of triage and separation between patients has confronted them with ethical dilemmas, particularly in the case of patients who are at risk of both a relapse of their mental illness and developing severe forms of COVID-19. Guidelines were sometimes developed to help identify patients for whom a face-to-face appointment was absolutely necessary [12]. Some authors warned against excluding vulnerable people or people living in poverty who do not have access to the internet or are limited by low digital literacy [63,68]. The situation of older adult patients, who are often unfamiliar with new technologies, also requires special attention. Not supporting them in the use of these tools constitutes a form of ageism [31]. Not only are older people not always averse to new technologies, but the issue of distance is all the more important as they are vulnerable to COVID-19 [65]. The experience of these new uses of digital technology opens the way to forms of differentiated care that make it possible to adapt care delivery to patients’ preferences, in order to improve the overall quality of care.

### Uses That Do Not Exploit the Full Potential of Digital Technology

In showing many professionals the potential of digital technologies, the COVID-19 crisis also revealed the extent to which their nonuse can be an ethical challenge. Digital technologies can reduce regional inequalities in access to health care [52] and can prevent significant costs and travel time [18,68]. Moreover, many patients have expressed their satisfaction with their experience of telepsychiatry [19,32,46,58], which confirms previous data from the literature [3]. Although some patients feel less supported in teleconsultations, others appreciate the freedom to access health care from an online platform [19]. Telepsychiatry can also foster patient autonomy [19,50,76] and the development of a form of empowerment that health organizations have been advocating for [77]. For example, it allows patients to record consultations in order to fully assimilate the information, thus increasing their power to act on the care relationship [50]. The experience of videoconferencing has allowed some professionals to overcome part of their initial reluctance [6] and to realize how valuable the virtual space of teleconsultation can be for building certain forms of intimacy in the therapeutic relationship [76,78]. However, in emergency and unpreparedness contexts, the experience of telepsychiatry did not take place in optimal conditions for their appropriation. In particular, some professionals experimented with videoconferencing when they were teleworking and not in their offices. The COVID-19 crisis has, nevertheless, made visible the extent to which digital technology can be a driving force for change in psychiatry [26]. It paves the way for the development of a hybrid care system integrating the strengths of teleconsultations as a complement to face-to-face encounters [3,8,76].

However, while the use of teleconsultation has been significant, professional uses of apps and connected devices do not seem to have been as stimulated by the health crisis. According to some authors, the pandemic, nevertheless, made it urgent to use such tools in order to intervene on a large scale to relieve the mental health burden induced by the crisis [54,67,79]. By providing clinical information, dedicated apps can also help to develop more personalized care plans [67]. The possibilities offered by digital phenotyping open up new perspectives for remote monitoring and assessment, making it possible, in particular, to detect the occurrence of disorders or relapses [48,49]. Even in a crisis context, the use of these tools has been hampered by the lack of both evaluations proving their effectiveness [54,64,79] and appropriate regulations [64]. Although the use of digital technologies has been stimulated by the crisis, in health care systems, it has essentially consisted of a transfer from in-person consultation to teleconsultation. In the end, health care has not taken full advantage of the specific intervention potential of digital technologies and artificial intelligence.

### Challenges to Better Integration of Digital Technologies in the Organization of Health Care

The COVID-19 health crisis questions the organization of care and the way it integrates new possibilities offered by digital technology. The scientific publications that we have identified mainly addressed the issues related to teleconsultation, sometimes ignoring previous research. The impact of the use of digital technology on relationships between professionals is poorly documented. However, the use of digital technologies is reshaping the conditions of teamwork and allows for new modalities of interprofessional collaboration [80,81]. The stakes are high, since digital technologies open up the possibility of numerous contacts between professionals working in different places. In particular, they make possible new collaborations between mental health and somatic professionals, and they allow a reorganization of consultation-liaison psychiatry. Constraints also need to be examined, since videoconference work meetings reduce interactional diversity, especially informal interactions. Such development is likely to affect well-being at work and social support between professionals in the context of crisis. However, other research has shown that telepsychiatry can allow professionals to optimize their working time and reduce their risk of burnout [73]. Remote team management, however, requires specific approaches [80].

Digital technologies also question the place of users and their relatives in the organization of care. As a result of the increase in outpatient and remote follow-up during lockdown periods, many patients have become more autonomous in managing their mental health [50]. Telepsychiatry can support the development of integrated patient-centered care, allowing for a more precise match between health care providers’ skills and patients’ needs [81]. Families have also been placed in the front line, pushed to take on new responsibilities [18,68]. Because community health services limited their travel, families relied more heavily on community resources [23]. New possibilities offered by digital technology thus invite new research to be conducted into community-based approaches to mental health.

### Conclusions

The COVID-19 pandemic has led to new uses of telepsychiatry, with the aim of ensuring continuity of care while limiting the risk of transmission of SARS-CoV-2. Such expansion was essentially characterized by the integration of videoconferencing as a new framework for consultation. Many mental health and psychiatric professionals started experiencing remote health care monitoring and assessment in a hurry and with no preparation. They have become familiar with the constraints, possibilities, and assets of care relationships in this type of context. These new conditions of professional practice have confronted them with ethical questions, such as equity in access to care. Existing research resources and data could be mobilized to enable these professionals to better leverage the benefits of digital technologies to complement face-to-face meetings. Further interdisciplinary work will be needed to better understand variations in digital technology uses across countries.

The use of digital technologies during the COVID-19 epidemic have shed light on the organization of mental health and psychiatric care, and about the place of users within this context of care. In a context where hospitals and health centers are no longer the only spaces where care and support are delivered, access to care and “decoding” the eHealth world constitute a pillar of tomorrow’s public health [71].

## Abbreviations

ARN: French National Research Agency
PHRC: French National Hospital Program for Clinical Research
PRISMA: Preferred Reporting Items for Systematic Reviews and Meta-Analyses
